# Too much of a good thing: a review of primary immune regulatory disorders

**DOI:** 10.3389/fimmu.2023.1279201

**Published:** 2023-10-31

**Authors:** Christo Tsilifis, Mary A. Slatter, Andrew R. Gennery

**Affiliations:** ^1^ Paediatric Immunology and Haematopoietic Stem Cell Transplantation, Great North Children’s Hospital, Newcastle upon Tyne, United Kingdom; ^2^ Translational and Clinical Research Institute, Newcastle University, Newcastle upon Tyne, United Kingdom

**Keywords:** hemophagocytic lymphohistiocytosis, immunodeficiency, immune dysregulation, HSCT, targeted therapies

## Abstract

Primary immune regulatory disorders (PIRDs) are inborn errors of immunity caused by a loss in the regulatory mechanism of the inflammatory or immune response, leading to impaired immunological tolerance or an exuberant inflammatory response to various stimuli due to loss or gain of function mutations. Whilst PIRDs may feature susceptibility to recurrent, severe, or opportunistic infection in their phenotype, this group of syndromes has broadened the spectrum of disease caused by defects in immunity-related genes to include autoimmunity, autoinflammation, lymphoproliferation, malignancy, and allergy; increasing focus on PIRDs has thus redefined the classical ‘primary immunodeficiency’ as one aspect of an overarching group of inborn errors of immunity. The growing number of genetic defects associated with PIRDs has expanded our understanding of immune tolerance mechanisms and prompted identification of molecular targets for therapy. However, PIRDs remain difficult to recognize due to incomplete penetrance of their diverse phenotype, which may cross organ systems and present to multiple clinical specialists prior to review by an immunologist. Control of immune dysregulation with immunosuppressive therapies must be balanced against the enhanced infective risk posed by the underlying defect and accumulated end-organ damage, posing a challenge to clinicians. Whilst allogeneic hematopoietic stem cell transplantation may correct the underlying immune defect, identification of appropriate patients and timing of transplant is difficult. The relatively recent description of many PIRDs and rarity of individual genetic entities that comprise this group means data on natural history, clinical progression, and treatment are limited, and so international collaboration will be needed to better delineate phenotypes and the impact of existing and potential therapies. This review explores pathophysiology, clinical features, current therapeutic strategies for PIRDs including cellular platforms, and future directions for research.

## Introduction

Primary immune regulatory disorders (PIRDs) are a group of inborn errors of immunity (IEI) defined by excessive inflammation, autoimmunity frequently targeting multiple tissues, lymphoproliferation, and malignancy, resulting from loss or gain of function in immunity-related genes associated with the regulatory mechanism of the inflammatory or immune response. Additionally, patients may be susceptible to severe, recurrent or opportunistic infection from impaired cellular or humoral immunity, from accumulation of end-organ damage, or from immunosuppressive therapy used to control immune dysregulation. In contrast, immune dysregulation covers a combination of autoinflammation, autoimmunity, and lymphoproliferation alongside susceptibility to severe infections, and can manifest by a number of different genetic conditions in which loss of the regulatory mechanism is not the primary defect. IEIs in which immune dysregulation may feature include, but are not limited to, chronic granulomatous disease and Wiskott-Aldrich syndrome. The distinction is important, because the dysfunction of a single molecule in PIRDs makes them potentially amenable to targeted therapy using small molecules or specific molecular inhibitors.

The diversity of clinical manifestations in PIRDs, which frequently present to non-immunologists, may lead to delayed recognition of the immunological and genetic diagnoses, and thus this patient group can pose a significant therapeutic challenge. Prompt recognition and molecular diagnosis is important to prevent multiple organ morbidities and side effects of prolonged immunosuppression, which underlie initial management for PIRDs; this is increasingly relevant as targeted therapies become available for specific molecular entities.

The number of genetically defined PIRDs has grown significantly as our understanding of mechanisms of immune tolerance, and access to genomic medicine has expanded. The concept of PIRDs as a disease entity was seeded from identification of the association between immunodeficiency and autoimmunity over 5 decades ago (1). This idea initially challenged understanding of IEI syndromes, given the apparent contradiction of excessive immune activity, demonstrated by autoimmunity and lymphoproliferation, yet recurrent infections and malignancy suggesting a degree of immunoparesis. It was recognized that even “classical primary immunodeficiencies” can manifest autoimmune features (2). Proposed theories to explain this included incomplete clearance of pathogens in immunodeficient patients causing a suboptimal, chronic immune response and thereby damage to ‘bystander’ tissues (2). In 1982, a case series describing a family where 17 male infants died of diarrhea associated with early-onset eczema and autoimmunity directed against multiple endocrine glands led to the description of the prototypical PIRD – immune dysregulation, polyendocrinopathy, enteropathy, X-linked (IPEX) syndrome (3) – and subsequent link with regulatory T-lymphocytes (Tregs) (4). Other disorders of this lymphocyte family constitute a subclassification of PIRDs. Many other PIRDs have since been described. Within the past decade, the number of PIRD genes listed in the International Union of Immunological Societies (IUIS) phenotypic classification of IEIs has grown from 21 (5) to 54 (6), shaping our understanding of immune function.

In this review, we discuss common manifestations of PIRDs, sitting at the crossroads of infection and autoimmunity, and the challenges of describing this heterogeneous group; disease subgroups including disorders of T regulatory lymphocytes, familial hemophagocytic lymphohistiocytosis (fHLH) syndromes, IEIs associated with very early onset inflammatory bowel disease, and diseases of autoimmunity and lymphoproliferation. We explore current and future directions for therapy such as targeted molecular treatment, allogeneic hematopoietic stem cell transplantation, and other cellular therapies.

## Pathophysiology of PIRDs

Immune dysregulation may occur from disorders of a number of mechanisms spanning the breadth of immune function. However, PIRDs arise specifically from disordered regulation of immunity and inflammation.

**Figure 1:**
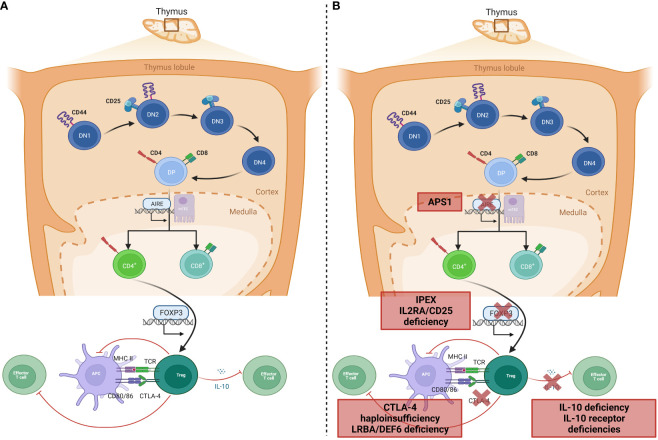
Genetic aetiology of specific PIRDs. **(A)** Normal T-lymphocyte development within the thymus results in removal of T-lymphocytes that bind self-antigen expressed by AIRE, on mTEC cells in the thymic medulla. A subset of CD4+ lymphocytes develop into Tregs with suppressor functions on effector T-lymphocytes through inhibition of co-stimulation (via CTLA-4 binding), or IL-10 production. **(B)** Monogenic defects of proteins involved in tolerance mechanisms of Treg development or function result in specific PIRDs. Adapted from “T-cell development in thymus 2” and “Tregs suppress dendritic cells and effector T cells”, by BioRender.com (2023). Retrieved from https://biorender.com/biorender-templates.

Instrumental to the prevention of autoimmunity in a healthy individual are the processes of central and peripheral immune tolerance. Central tolerance occurs within the thymus: during T-lymphocyte development, progenitor T-lymphocytes migrate from the bone marrow to the thymic cortex to undergo proliferation, maturation, rearrangement of their T-cell receptors (TCR), and, in the thymic medulla, differentiation into mature T-lymphocytes that may enter the peripheral circulation (**Figure 1A**). These mature T-lymphocytes must be capable of recognizing and reacting to pathogens, virus-infected cells, and malignant cells through binding of antigen to the TCR, whilst crucially being tolerant to self-antigens; this is central tolerance. Central tolerance develops through two sequential stages, whereby immature double-negative (DN) T-lymphocytes that express the CD3-TCR complex but lack either CD4 or CD8 interact with thymic cortical epithelial cells to positively select TCRs that bind to the major histocompatibility receptor I and II expressed by ‘self’ cells, or else undergo apoptosis; this is termed positive selection, leading to a population of CD4+CD8+ (double positive, DP) lymphocytes. DP lymphocytes undergo negative selection in the thymic medulla, where lymphocytes reactive to self-antigens expressed by medullary thymic epithelial cells (mTEC) are deleted (7). Expression of this restricted set of tissue antigens by mTECs relies on the transcription factor autoimmune regulator, encoded by the *AIRE* gene (8). Impairment of central tolerance by inherited defects of thymic development leads to syndromes including the autoimmune lymphoproliferative syndromes (ALPS), caused by failure of the extrinsic activation-induced cell death pathways (mutations in *FAS*, *FASLG*, and *FADD*), and autoimmune polyglandular syndrome (APS) 1, caused by mutations in *AIRE*.

Once single-positive mature T-lymphocytes expressing either CD4 or CD8 are in the peripheral circulation, the effector functions are checked by Tregs, which maintain peripheral tolerance by upregulation of cell-suppressing surface markers such as cytotoxic lymphocyte antigen-4 (CTLA-4) and production of inhibitory cytokines including IL-10 (9, 10). The repertoire of Tregs extends beyond peripheral tolerance, and includes suppression of allergic inflammation, induction of tolerance to dietary antigens and to the fetus during pregnancy, and protection of the microbiome (11). It is therefore unsurprising that Tregopathies frequently feature an allergic preponderance. Tregs develop in the thymus as natural Tregs (nTregs) or following antigen exposure peripherally as inducible Tregs (iTregs). Treg development and function is dependent on the forkhead box protein-3 (FOXP3) transcription factor, deficiency of which abrogates Treg development and causes IPEX syndrome with absent or dysfunctional Tregs (9) (**Figure 1B**). A phenotypically similar syndrome is caused by deficiency of CD25 (the α-chain of the IL-2 receptor) (12, 13), which is universally expressed on Tregs. Functional Treg defects are seen in defects of surface markers and intracellular proteins such as CTLA-4 (14) and the closely-related lipopolysaccharide-responsive beige-like anchor (LRBA, **Figure 1B**) (15). Additionally, dysfunction of suppressor cytokine pathways involving IL-2 (15, 16), IL-10 (17) either by deficiency of receptors or altered downstream signaling molecules such as JAK1 (18), STAT1 (19), STAT3 (20), and STAT5b (21), renders ineffective the common mechanisms by which Tregs usually restrain inflammation.

PIRDs may also manifest in hyperactivation or hyperinflammation that functioning peripheral tolerance mechanisms cannot restrain: this typically manifests as HLH, a syndrome of fever, hepatosplenomegaly, and consumption of hematopoietic-derived cells in the peripheral blood, bone marrow and central nervous system (hemophagocytes) due to excessive pro-inflammatory cytokine release from activated macrophages. This process is usually prevented by the inhibitory effect of NK and CD8+ lymphocytes, which insert pores into macrophage cell membranes to deliver cytotoxic granules and cause cell death, through the intrinsic cell death pathway. Monogenic defects in pore formation, cytotoxic granule production, and their delivery cause familial HLH (22). However, a range of additional immune disorders may predispose to HLH due to NK cell deficiency or impairment, suboptimal handling of viral infections such as EBV, or dysregulated inflammasome control. HLH seen in the context of these other IEI is termed primary HLH (23).

## Common manifestations of PIRDs

PIRDs pose a diagnostic and therapeutic challenge for clinicians due to the broad spectrum of potential manifestations involving multiple organs, which may therefore present to various medical specialities. Autoimmunity may be antibody- or cell-mediated, and can target hematopoietic cells causing cytopenias, endocrine organs causing type 1 diabetes mellitus (T1DM) or thyroiditis, skin and connective tissue causing dermatitis, vitiligo, alopecia or arthritis, visceral organs causing hepatitis or glomerulonephritis, and the epithelial barrier causing uveitis or inflammatory bowel disease. Individually, these manifestations may be common: immune thrombocytopenia purpura has an estimated incidence of 1.9-6.4 per 100,000 children each year (24), whilst T1DM has an incidence of 22.9/100,000 per year (25). This makes early diagnosis of potential PIRDs difficult until multi-organ autoimmunity develops, or unless additional features such as a positive family history or severe or recurrent infection are present. Allergic disorders such as food or drug allergy, rhinitis, and asthma may also manifest. Impaired control of lymphoid cells may interact with abnormal handling of viruses such as Epstein-Barr virus (EBV) and predispose to lymphoproliferation and development of lymphoma.

Given the multi-organ nature of many PIRDs, understanding and quantifying the burden of immune dysregulation is important, and may be done through disease activity scoring systems. At an individual patient level, these scoring systems allow clinicians to track response to therapies, whilst at a population level, they aid understanding natural history and progression of different monogenic diseases. The heterogeneity between different PIRDs and failure to identify a monogenic defect in every patient with immune dysregulation features makes such scoring systems challenging to design. The Immune Deficiency and Dysregulation Activity (IDDA) score is a composite of the presence and severity of different clinical parameters, graded 0–4 depending on the impact of each manifestation. It is altered by other factors such as chronic infection and duration of hospitalization. This was initially conceived to provide a comparison of phenotype in patients before and after HSCT for LRBA deficiency (26), but has since been updated to encompass all PIRDs (IDDA2.1 (27)). It can be used to longitudinally track disease activity in individual patients and assess response to treatment, and used visually represent different disease phenotypes through a ‘kaleidoscope’ function (27).

## Moving towards targeted therapy for PIRDS

Historically, challenges in obtaining a molecular diagnosis for PIRDs has led to patients receiving prolonged immunosuppression, particularly with corticosteroids. Chronic corticosteroid use carries significant morbidity due to weight gain, risk of osteoporotic fractures, and immunosuppression; the latter may be particularly important in these diseases where there is infection susceptibility in addition to autoimmunity.

Allogeneic HSCT may be considered diametrically opposed to targeted therapy; rather than selecting a specific pathway to modulate, HSCT offers the opportunity to replace recipient stem cells carrying a disease-causing allele with those from a healthy donor, thereby affecting all bone marrow-derived cells. The benefits of HSCT must be balanced against the limitations of its use: firstly, HSCT may not correct the underlying defect in all PIRDs, particularly in cases where there is pleiotropic expression of the mutated protein (such as in STAT3-GOF), or where the defect is of thymic origin (such as APS1). Secondly, HSCT carries a significant and variable risk of mortality. This is typically associated with early transplant-related complications arising from preparative conditioning, from the period of aplasia prior to engraftment of donor cells and subsequent immune reconstitution, and from alloreactivity from donor lymphocytes causing graft-versus-host disease (GvHD). Thirdly, HSCT may not be available in every healthcare setting, particularly when considering the differences between HSCT experience in IEI patients as opposed to more common indications such as hematological malignancy, and even if available, is expensive and reliant on availability of a well-matched donor. These limitations in availability have led to the subset of patients undergoing HSCT in historic series representing a multi-morbid, therapy-resistant cohort who were transplanted without a genetic diagnosis, late in their disease course with accumulated end-organ damage and treatment side effects. An additional challenge in HSCT for PIRDs as opposed to ‘classical’ immunodeficiency is the need to attain sufficient myeloablation to remove recipient lymphocytes which may facilitate graft rejection, whilst minimizing associated toxicities. A degree of alloreactivity is also required so that hyperactivated recipient cells may be destroyed by emerging donor immunity. HSCT for PIRDs thus represents the intersection of philosophies for treatment of hematological malignancy, where conditioning regimens favor myeloablation and where alloreactivity may aid a graft-versus-leukemia effect, and of treatment of classical immunodeficiency, where pre-HSCT infection and organ-damage is common and conditioning intensity aims to minimize associated toxicity. Together, these challenges result in poorer HSCT outcomes for PIRDs than for other monogenic IEIs such as chronic granulomatous disease or Wiskott-Aldrich syndrome, with particularly high rates of graft rejection or GvHD (28, 29). The role of autologous *ex-vivo* gene therapy for these diseases is evolving; whilst successful engraftment of corrected HSCs still mandates conditioning to create a marrow ‘niche’, advantages include ability to harvest from the patient and not rely on a matched donor, and no risk of GvHD (30). Gene therapy platforms have been established and trials are in clinical phases for severe combined immunodeficiency (SCID; *IL2RG*, *RAG1, DCLRE1C*) and non-SCID IEI including IPEX, Wiskott-Aldrich syndrome, and X-linked chronic granulomatous disease (30, 31). Gene therapy trials for familial HLH genes (*UNC13D*, *PRF1*) are currently in pre-clinical phases (32, 33). Whilst attractive, application of this science to the clinical setting is in early stages, with little long-term outcome data and concerns regarding cost and availability (34).

The ability to maintain remission of autoimmunity with minimal side-effects is therefore an attractive alternative to both chronic corticosteroid use, and allogeneic HSCT. Today, with several agents being either disease mechanism-specific (such as abatacept) or pathway-specific (such as JAK inhibitors), control of underlying immune dysregulation may be better balanced against side-effect profile, and thus the treatment paradigm may be changed. Diseases affecting CTLA-4 expression (CTLA-4 haploinsufficiency and LRBA/DEF6 deficiencies) may be treated with replacement of CTLA-4 protein in the form of abatacept or belatacept, with good clinical response, particularly for enteropathy or lymphoid cell-infiltrative disease of the lungs or nervous system (35, 36). A multicenter trial exploring its efficacy and safety is currently underway (37). For several diseases where there is a quantitative or qualitative Treg defect and subsequent hyperactivation of effector T lymphocytes, mTOR inhibition using sirolimus has proven to be an effective steroid-sparing agent (35, 38–40). It may also be useful in ALPS (41). A range of agents antagonizing common pro-inflammatory mediators such as IL-1 (anakinra) and IFN-γ (emapalumab) may be employed to aid remission in primary HLH, whilst specific aetiologies where IL-18 hyperactivation is implicated may benefit from IL-18 binding protein analogues (42–45). B-lymphocyte directed therapy, such as rituximab, may benefit diseases where EBV infection causes HLH (such as SAP and XIAP deficiencies) or where autoantibody production is problematic (such as APS1, or CTLA-4 haploinsufficiency) (35, 46). Anakinra, which is a recombinant IL-1 receptor antagonist, may remit the multi-system inflammation seen in disease states where IL-1 antagonism is impaired such as deficiency of endogenous IL-1RA and the closely-related IL-36RA (DIRA and DITRA syndromes, respectively) (47, 48). For PIRDs caused by hyperactivation of a specific protein, use of small molecules to inhibit activity and return it to baseline is attractive and has been trialed for activated phosphoinositide 3-kinase-δ syndrome (49). Given the broad clinical and immune phenotype of PIRDs, and that individual diseases may cause symptoms through different pathways, it is unsurprising that there is no ‘silver bullet’ that can remit all aspects of a disease. Furthermore, the novelty of several of these agents and small patient population means long-term data on efficacy and safety are limited, and the financial and clinical impact of very long-term treatment is yet to be determined. Collection of ongoing longitudinal data is required to fully understand whether these treatments will serve as long-term maintenance therapy, a tool for inducing remission, or a bridge to definitive cure with HSCT or other cellular therapies.

## Specific disease groups

We will discuss specific PIRD entities, defined either by genotype or by phenotype, and their specific clinical and immunological characteristics and current understanding of different treatments. **Table 1** summarizes disease entities and strategies for treatment, including targeted therapy and results of HSCT or gene therapy.

**Table 1:** Summary of PIRDs discussed in this review, with genetic and clinical features, and options for treatment including mechanism-specific therapies, HSCT, and gene therapy.

Disease Group	Disease	Gene and inheritance	Pathophysiology	Clinical features	Therapeutic options	Targeted therapy	HSCT	Gene therapy
Familial HLH	fHLH2	*PRF1*; AR	Impaired cytotoxic granule formation	Primary HLH Enteropathy in fHLH5	HLH2004 (etoposide, dexamethasone, ciclosporin A ± intrathecal methotrexate) Alemtuzumab	Anakinra (IL-1 receptor antagonist) Emapalumab (anti-IFN-γ) Ruxolitinib (JAK-inhibitor)	OS 71%	Pre-clinical trials (50)
	fHLH3	*UNC13D*; AR	Impaired vesicle trafficking					Pre-clinical trials (33)
	fHLH4	*STX11*; AR	Impaired vesicle docking and fusion					–
	fHLH5	*STXBP2*; AR						–
Other IEI associated with primary HLH	XLP-1	*SH2D1A*; XL	Impaired SAP-dependent T and NK cell cytotoxicity	Hypogammaglobulinemia Lymphoproliferation → lymphoma EBV-triggered HLH	Ig replacement	Rituximab (anti-CD20)	OS 35/43 (81.4%), but only 50% if preceding HLH (51)	Pre-clinical trials (52)
	XIAP deficiency	*XIAP*; XL	Inappropriate inflammasome activation → high IL-18	EBV-triggered HLH Inflammatory bowel disease	HLH: HLH2004 IBD: steroids, rituximab, infliximab, alemtuzumab	Tadekinig alfa (under investigation)	OS 35/50	–
	NLRC4 GOF	*NLRC4*; AD					Not reported	–
Defects of central tolerance	APS1	*AIRE*; AR but some heterozygous mutations cause a milder phenotype	Defect in central tolerance → auto-reactive lymphocyte escape	Candidiasis Multi-organ autoimmunity including endocrinopathy (adrenal insufficiency, hypoparathyroidism) pneumonitis, hepatitis Urticarial eruption Malabsorption	Azathioprine MMF	Sirolimus (mTOR inhibitor) Rituximab (anti-CD20)	Does not correct	–
ALPS and ALP-like	ALPS-FAS ALPS-FASL ALPS-CASP10	*FAS*; AD *FASL*; AR *CASP10*; AR	Peripheral defect in extrinsic programmed cell death pathway → accumulation of auto-reactive lymphocytes	Lymphoproliferation → lymphoma AIC Multi-organ autoimmunity including eczema, alopecia, hepatitis, vasculitis	Immunoglobulin Steroids	Sirolimus (mTOR inhibitor)	Reported, but typically not required (53)	–
	ALPS-FADD	*FADD*; AR		As above, plus infection/vaccination-provoked encephalopathy, pneumococcal sepsis			N=2; successful in both patients (54)	–
Tregopathies	IPEX	*FOXP3*; XL	Abrogated Treg development	Endocrinopathies such as neonatal diabetes mellitus Eczema Enteropathy Nephrotic syndrome	Steroids Calcineurin inhibitors	Sirolimus (mTOR inhibitor)	OS 43/58 (73.2%), better if lower organ involvement score (55)	First-in-human trials (31)
	IL-2RA/CD25 deficiency	*IL2RA*; AR	Impaired Treg function	IPEX-like, plus CMV susceptibility	Steroids Infliximab	Sirolimus (mTOR inhibitor)	N=1; successful (56)	–
	IL-2RB/CD122 deficiency	*IL2RB*; AR	Impaired Treg number				N=3; successful in two patients (57)	–
	STAT3-GOF	*STAT3*; AD	Enhanced STAT3 and diminished STAT1/STAT5 phosphorylation Raised IL-6	Childhood-onset lymphoproliferation and autoimmunity; AIC, growth delay, enteropathy, vasculopathy, malignancy	Steroids MMF	Tocilizumab (anti-IL-6) Ruxolitinib (JAK inhibitor)	OS 15/23 (62%) (58)	–
	CTLA-4 haploinsufficiency	*CTLA4*; AD	Enhanced T-lymphocyte activation due to loss of CTLA-4 checkpoint	Lymphoproliferation → lymphoma, gastric cancer Lymphoid infiltration of CNS, lungs, GI tract Endocrinopathy, AIC, hepatitis, nephritis	Steroids MMF Azathioprine	Abatacept, belatacept (CTLA-4 fusion protein) Rituximab (anti-CD20) Sirolimus (mTOR inhibitor)	OS 15/21 (71.4%) (35)	Proof of concept (59)
	LRBA deficiency	*LRBA*; AR					OS 17/24 (70.8%) (26)	–
	DEF6 deficiency	*DEF6*; AR					Not reported	–
IL-10-related VEOIBD	IL-10 deficiency IL-10 receptor deficiency	*IL10;* AR *IL10RA;* AR *IL10RB;* AR	Defect in IL-10 production or receptor formation → intestinal inflammation	VEOIBD with perianal anal disease Folliculitis, arthritis	Steroids Azathioprine Infliximab	Anakinra (IL-1 receptor antagonist)	OS 5/5 (60)	–
APDS	APDS1 APDS2	*PI3KCD;* AD *PI3KR1*; AD	Altered T- and B-lymphocyte homeostasis, dysgammaglobulinemia	Sinopulmonary infection → bronchiectasis Lymphoproliferation and chronic EBV infection, lymphoma Enteropathy, AIC, dermatitis	Steroids Immunoglobulin replacement	Rituximab (anti-CD20) Sirolimus (mTOR inhibitor) Leniolisib (PI3Kδ inhibitor)	OS 49/57 (86%) (61) High rates of graft failure, particularly if mTOR inhibitors used post-HSCT	–
Predominately autoinflammatory syndromes	DNase1 deficiency DNase1L3 deficiency	*DNASE1;* AD *DNASE1L3*; AR	Impaired clearance of chromatin and double-stranded DNA → autoantibody formation	Systemic lupus erythematosus	Steroids Other disease-modifying anti-rheumatic drugs	Rituximab (anti-CD20)	Not reported	–
	Haploinsufficiency of A20 (HA20)	*TNFAIP3;* AD	Enhanced NFκβ signaling → upregulation of pro-inflammatory cytokines	Behcet-like disease with orogenital and gastrointestinal ulceration, uveitis, arthritis Fever	Steroids Colchicine	Infliximab, adalimumab (anti-TNFα) Tociluzumab (anti-IL-6)	OS 2/2 (62)	–
	IL-1RA deficiency (DIRA) IL-36RA deficiency (DITRA)	*IL1RN;* AR *IL36RN;* AR	Reduced antagonism of IL-1 → enhanced pro-inflammatory cytokine activity via NFκβ	Sterile osteoarticular inflammation (only described in DIRA) Recurrent fever Pustulosis and generalized pustular psoriasis	Steroids	Anakinra (IL-1 receptor antagonist)	Not reported	–

### APS1/APECED

Biallelic LOF mutations in *AIRE*, which are autosomally inherited, cause a defect in central tolerance with failure to delete auto-reactive lymphocytes in the thymus, manifesting in a syndrome characterized by chronic mucocutaneous candidiasis and multi-organ autoimmunity, particularly against parathyroid and adrenal glands (autoimmune polyendocrinopathy-candidiasis-ectodermal dystrophy, APECED, also called APS1) (63). The syndrome was first reported in 1929 (64), with its genetic aetiology identified by two groups in 1997 (65, 66), and has a prevalence that varies by population, with certain founder mutations found in relative high numbers of the Persian Jewish, Sardinian, and Finnish populations (67). There is poor genotype-phenotype correlation, with significant intra-familial variability despite sharing identical *AIRE* mutations (68). However, some features appear enriched within an American cohort of patients, particularly a non-pruritic urticarial eruption which, in this cohort, was as prevalent as CMC. The diagnostic criteria therefore expanded to also include urticarial eruption, enamel hypoplasia, and intestinal malabsorption (69). Recently, heterozygous *AIRE* mutations exerting negative dominance have been identified in a syndrome with milder autoimmunity and incomplete penetrance (70).

Whilst the classic triad of hypoparathyroidism, Addison’s disease and CMC define the syndrome, the phenotype has expanded in recent years to include other endocrine autoimmunity impacting pancreatic islet cells, thyroid, and gonad; visceral autoimmunity including pneumonitis, hepatitis, gastritis and intestinal malabsorption; non-infectious epithelial features such as urticarial eruption, alopecia, vitiligo, nail dystrophy and enamel hypoplasia; and a Sjogren’s-like sicca syndrome (67). Whilst the infective manifestations have classically been restricted to epithelial-site Candida infection only, the recent emergence of the SaRS-CoV-2 pandemic and subsequent discovery of anti-type-I interferon antibodies underlying mortality risk from pneumonitis in both APECED patients (71) and the general population (72). Some patients develop epithelial-site malignancy associated with chronic Candida infection.

These infective complications relate to autoimmunity: failure of AIRE to project an “immunological self-shadow” (73) within the thymus causes escape of auto-reactive T lymphocytes into the circulation, which then infiltrate organs and cause autoimmunity either by a direct cellular effect, or by induction of autoantibody formation, possibly in tandem with auto-reactive B lymphocytes (46, 67). Multiple autoantibodies have been associated with APECED features, including against NACHT leucine-rich-repeat protein 5 and the calcium-sensing receptor (hypoparathyroidism), 17-α and 21-hydroxylases (adrenal insufficiency), intrinsic factor (pernicious anemia), and cytokines such as the IL-17 family (CMC), IL-22, and type-I interferons -α, -β, -λ, and -ω (SaRS-CoV-2 pneumonitis) (74); interestingly, anti-interferon-α antibodies have been posited to provide protection from development of T1DM in some APECED patients (75). The *AIRE* defect may also impair Th17 lymphocyte development, possibly explaining the Candida diathesis in APECED patients without detectable IL-17 autoantibodies (76).

Treatment of APECED commonly involves specialists from multiple disciplines including endocrinology, immunology, gastroenterology/hepatology and dermatology. CMC may be managed with azole antifungals, or amphotericin B in cases of azole resistance; patients should be screened for asplenia, which may co-occur in ~10% of patients. Autoimmunity may be managed with azathioprine, mycophenolate mofetil, or mTOR inhibitors such as sirolimus; B-lymphocyte depletion with rituximab has been described in cases of recalcitrant autoantibody-mediated organ disease and in pneumonitis (77). Allogeneic HSCT does not correct this disease, which is caused by a thymic defect.

### ALPS and ALP-like syndromes

Autoimmune lymphoproliferative syndrome (ALPS) and ALP-like syndromes are defects of lymphocyte homeostasis caused by impairment of the FAS-mediated apoptosis pathway. Classical ALPS was first described in 1967 and given the eponym Canale-Smith syndrome after the paper’s authors (78), as a syndrome of non-malignant lymphoproliferation with autoimmunity. Subsequently, a predisposition to lymphomatous transformation was also identified, and the syndrome was associated with deleterious variants in *FAS (* 79) followed by *FASL (* 80), *CASP10 (* 81) and somatic *FAS (* 82) mutations. These genes encode the proteins of the FAS-mediated apoptosis pathway: FAS, a cell-surface receptor, binds to its ligand (FAS-L) leading to conformational change and recruitment of intracellular proteins (caspase-8 and caspase-10, and FAD-associated death domain) to herald programmed cell death. This process is upregulated in T-lymphocytes which react to self-antigen, restraining proliferation of autoreactive lymphocytes: thus, failure of this mechanism causes an accumulation of autoreactive double-negative (TCRαβ+CD4-CD8-) T-lymphocytes (DNTs) with a significant proliferative potential (83). Along with clinical features and DNTs, biomarkers such as soluble FAS-L, vitamin B12, and IL-10 comprise the diagnostic criteria for ALPS (83).

Mutations in *FAS* are most commonly dominant and in the germline, with somatic mutations representing 15-20% of cases. However, some *FAS* mutations behave in a recessive manner, with heterozygous probands being minimally symptomatic. Similarly, the much rarer ALPS-FAS-L usually requires a biallelic mutation to cause disease (83, 84). The incomplete clinical penetrance of ALPS may be partially explained by a ‘double hit’ being necessary to impair FAS-related apoptosis (85). Patients lacking a mutation in the FAS-associated genes are categorized as ALPS-U; advances in availability of high-throughput genomic testing have identified mutations in >20 distinct IEI genes in ALPS-U patients, including other PIRDs such as Tregopathies (STAT3-GOF, CTLA-4 haploinsufficiency, LRBA deficiency) (86), highlighting how rapidly this field is expanding.

Clinical manifestations of ALPS typically begin in early childhood. Lymphoproliferation of DNTs causes multi-focal lymphadenopathy, particularly of cervical nodes, and splenomegaly. These demonstrate DNT infiltration and may remit in adulthood. Autoimmunity in ALPS is commonly directed against erythrocytes, platelets and neutrophils, with >80% of patients manifesting with hematological cytopenias (84). Organ-specific autoimmunity caused by autoantibody formation, possibly by disturbed B-lymphocyte homeostasis, may include uveitis, eczema, alopecia, hepatitis, Guillain-Barre syndrome and vasculitis (84); patients frequently have clonal IgG expansion. Lymphomatous transformation may occur, particularly into Hodgkin-type (87). Historically, splenectomy has been trialed to reduce the burden of lymphoproliferation; however, the risks of encapsulated bacterial infection compounded by attenuated polysaccharide vaccine responses do not support this treatment strategy (88). Autoimmune cytopenias (AICs) may respond in the first instance to immunomodulation with high dose intravenous immunoglobulin (1 – 2g/kg), though is commonly refractory and requires second-line therapy. Lymphoproliferation and autoimmunity may both respond to immunosuppression, typically steroids though mTOR inhibitors such as sirolimus have shown favorable results with minimal infection risk, possibly due to this pathway being hyperactivated in ALPS (40, 41). Following induction of remission, maintenance therapy may be continued to prevent relapse. In rare cases, due to uncontrolled life-threatening lymphoproliferation, HSCT may be considered but experience is limited to few reports (53, 88, 89). In a systemic review collating 12 patients with *FAS* or *FASL* mutations and ALPS who underwent HSCT, survival was seen in 11/12 (91.7%) (89). Transplant course was complicated by graft rejection necessitating second HSCT in two patients, and graft-versus-host disease (89).

Syndromes caused by mutations in caspase-8 (*CASP8*) or FAD-associated death domain (*FADD*), previously categorized as ALPS, behave differently. Caspase-8 deficiency may cause very early-onset inflammatory bowel disease (VEOIBD) presenting before age 6 years or other infiltrative autoimmune disease, contrasting with ALPS, or infection susceptibility (90, 91). FADD deficiency overlaps ALPS with the addition of infection- or vaccination-provoked encephalopathy and seizures, ocular findings, and overwhelming pneumococcal sepsis (54, 92–94). HSCT has been successful in 2 FADD-deficient patients (54).

### IPEX

Mutations in the *FOXP3* gene, on the X chromosome, cause IPEX syndrome with abrogated Treg development. This syndrome typically presents in the first few weeks of life with intractable diarrhea, enteropathy and resultant failure to thrive, eczema, and T1DM (3), though this triad may only occur in ~60% of patients (55, 95); other features include food allergies, nephrotic syndrome, other endocrinopathies, AIC and serious infections such as sepsis and meningitis. Immunologically, patients have low or absent Tregs with raised IgE and eosinophilia, and characteristic autoantibodies against renal and gut epithelial proteins (96, 97). The impact of mutation type and site on FOXP3 protein expression vary; mutations that confer small changes to the protein with intact expression appear to result in a milder phenotype (95). Certain phenotypes and outcomes cluster to specific FOXP3 protein domains, such as poorer outcome in repressor domain mutations (98), but there is significant extra-familial variability amongst patients with the same variant, suggesting influence of other genetic or environmental factors on phenotype. More recently, emphasis has been placed on atypical presentations of IPEX: these include intrauterine or late onset, mild course, or IPEX-like syndromes in patients with some features of the syndrome but no *FOXP3* mutation. Intrauterine IPEX may present with hydrops, ultrasonographic features such as hyperechoic bowel, and poor perinatal outcome including stillbirth or neonatal mortality (99), and appears to cluster in families. Relatively late-onset IPEX may present towards the end of the first decade of life with an IBD-like phenotype (100). Reports of mild IPEX with either single-organ involvement, or rapid response to immunosuppression, have been associated with non-coding region mutations and near-intact Treg compartments (100). In IPEX-like patients with intact FOXP3 function, mutations in other IEI genes such as *LRBA*, *STAT3* (GOF), *CTLA4*, *IL2RA* and *STAT5B* have been implicated (95).

Treatment of IPEX centers on nutritional support and immunomodulation such as steroids and adjunctive therapy, usually calcineurin inhibitors or sirolimus; the latter may offer better disease control by restoring Treg function (38). Retrospective data highlight that whilst immunosuppression may ameliorate the disease, it unsurprisingly does not reverse end-organ damage and may only temporize accrual of disease manifestations, particularly as follow-up extends into the second or third decade after diagnosis (55). Allogeneic HSCT offers a better disease-free survival than immunosuppression alone, though the risk of mortality (~25%) with HSCT is stratified by pre-HSCT morbidity, suggesting that prompt treatment or aggressive optimization of patient condition is critical for this treatment modality. Interestingly, mixed rather than full donor chimerism does not negatively affect outcome and leads to functional donor Treg production, suggesting that reduced intensity conditioning strategies may provide a balance between transplant-related morbidity and immunological outcome (55). A phase 1, first-in-human trial of restoration of the Treg compartment by induction of CD4+ cell development into Tregs following lentiviral transfer of wild-type *FOXP3* is currently in recruitment (NCT05241444) (31).

### CTLA-4 haploinsufficiency and LRBA and DEF6 deficiencies

First identified in humans in 2014, autosomal dominant mutations in *CTLA4*, causing haploinsufficiency of its encoded protein, cause a complex PIRD with incomplete penetrance (14, 101). CTLA-4, expressed on Tregs, acts as an immune checkpoint by outcompeting CD28 for binding to CD80/CD86 on antigen presenting cells, prompting transendocytosis of the complex and thereby terminating the co-stimulatory second signal required for T-lymphocyte activation (102). Following the first description in seven patients from four kindreds, understanding of CTLA-4 haploinsufficiency has expanded by publication of international, multicenter cohorts (36). Whilst mutations may not be completely penetrant, leading to divergent or absent symptoms within families sharing the same mutation, affected patients commonly have multiorgan involvement with lymphoid infiltration into lungs, the central nervous system, and gastrointestinal tract along with autoimmune endocrinopathies including diabetes, arthritis, cytopenias, and infections (36). Lymphoproliferation may be symptomatic in its own right, or transform into lymphoid malignancy, often driven by EBV, with a lifetime risk of approximately 1 in 6 patients (103, 104). Penetrance of CTLA-4 haploinsufficiency appears to be independent of the specific mutation (35). Patients may develop primary hypogammaglobulinemia as CTLA-4 is involved in differentiation of follicular helper T-lymphocytes, and this may be compounded by immunosuppression (36, 105). CXCR5+PD1+ follicular helper T-lymphocytes may act as a biomarker for CTLA-4 disease; other immunophenotype changes appear to be variable across the CTLA-4 haploinsufficient population (106).

These clinical features are shared with three other genetic diseases. Monoallelic deletions of the 2q33.2 – 2q33.3 locus, where *CTLA4* sits, present as phenotypic CTLA-4 haploinsufficiency (107). Recessive mutations affecting the lipopolysaccharide responsive beige-like anchor protein (*LRBA*) were identified in 2012 prior to CTLA-4 haploinsufficiency, in kindreds with childhood-onset hypogammaglobulinemia, impaired B-lymphocyte compartments, and autoimmunity (15). The overlap between these two diseases is explained by the role of LRBA in protecting CTLA-4 from lysosomal degradation, and indeed LRBA deficiency causes reduced cell-surface CTLA-4 expression (108). Also impacting CTLA-4 surface trafficking, homozygous mutations in *DEF6* impair regulation of the GTPase RAB11 and thereby reduce the intracellular trafficking of CTLA-4-containing vesicles towards the cell-surface (109, 110). Initially conceived for LRBA deficiency, a multiorgan scoring system (IDDA) may be used for assessing longitudinal response to therapy (26). Following its use in a cohort of 76 patients with LRBA deficiency, the IDDA score has been revised to incorporate other features of immune dysregulation such as hemophagocytes (IDDA2.1), and its inclusion in the ESID registry will help expand its use in describing the phenotype of PIRDs beyond LRBA deficiency (27).

Treatment for CTLA-4 haploinsufficiency and related disorders centers on immunomodulation. There is variability in organ response to different therapies. Corticosteroids are effective in remitting granulomatous lymphocytic interstitial lung disease and gastrointestinal inflammation, they appear less effective for neurological involvement. Steroid-sparing agents, such as mTOR inhibitors, TNFα inhibitors, and B-lymphocyte directed therapy with rituximab are commonly employed to provide multimodal immunosuppression and reduce the adverse effects associated with chronic corticosteroid use. The aetiology of immune cytopenias likely relates to marrow infiltration by autoreactive T-lymphocytes, rather than peripheral sequestration or destruction, explaining the poor clinical response following splenectomy (no response in 10/14 patients) (35). Immunoglobulin replacement effectively reduces infection frequency. Replacement of insufficient CTLA-4 in these diseases, by intravenous or subcutaneous infusion of the CTLA-4 fusion proteins abatacept or belatacept, may be considered (26, 35, 111). Response rates appear promising, particularly for neurological and gastrointestinal disease, although existing tissue destruction such as that causing insulin-dependent diabetes mellitus cannot be reversed. Long-term efficacy and safety data are lacking. Surveillance for oncogenic viruses such as EBV may be important, as chronic viraemia is common, malignancy is frequently EBV-related, and there have been reports of herpesvirus reactivations with CTLA-4 fusion protein therapy (103, 112, 113). A phase IIa prospective multicenter trial (ABACHAI) aims to answer questions surrounding safety and efficacy in adult patients with CTLA-4 haploinsufficiency by use of a novel morbidity score (CHAI), which could also be employed in future study of other therapies in this disease (37). Whilst HSCT has a role in these diseases, further data to understand the true extent of its efficacy and which patients should be transplanted, and when, are required. For LRBA deficiency, twenty-four patients underwent HSCT with a median follow-up of 20 months and OS of 70.8%, with all deaths being transplant related. Interval to HSCT and disease burden, particularly pulmonary involvement, affected odds of survival (26). Surviving patients had significantly lower IDDA scores than those on conventional therapies, and 70.6% were in immunosuppression-free remission. In CTLA-4 haploinsufficiency, two HSCT series were updated in a large multicenter study recently, and along with one other series a total of 21 transplanted patients have been reported in the literature, with OS 15/21 (71.4%) (35, 36, 114, 115). Whilst disease activity appears improved in HSCT survivors, donor Tregs do not appear to have the same survival advantage seen in HSCT for IPEX and consequently, immune dysregulation may continue in the setting of mixed chimerism (114, 116). Questions regarding optimum HSCT strategy, and the role of CTLA-4 fusion therapy as a bridge to transplant, may be answered by an ongoing study of the Inborn Errors Working Party of EBMT. Autologous gene therapy using a homology-directed repair approach in human *CTLA4* ^+/-^ CD4+ lymphocytes has successfully restored CTLA-4 activity in an *in vitro* study with good efficiency, but requires translation to an HSC platform (59).

### STAT3-GOF

Whilst dominant negative mutations in signal transduction and activator of transcription (STAT) 3 cause a complex multisystem immunodeficiency with recurrent staphylococcal and fungal infection, eczema, and connective tissue disease (STAT3 hyper IgE syndrome) (117, 118), germline activating mutations lead to exaggerated transcriptional activity of STAT3 and impaired signaling through the other STAT molecules (20). STAT3 transduces multiple cytokine signals, including IL-6, IL-10, and IL-23 (119, 120). The resultant immunological phenotype of STAT3-GOF is of a quantitative and qualitative Treg defect. Clinically, patients display an incompletely-penetrant phenotype of childhood-onset multiorgan autoimmunity and lymphoproliferation, along with stunted growth (20, 21, 121). Whilst the most common manifestations are lymphoproliferation with raised DN T-lymphocytes, two-thirds have autoimmune cytopenias and end-organs affected by autoimmunity include the gastrointestinal tract, endocrine organs, liver causing hepatitis, and lung disease. The immune deficiency was initially reported as modest, though a large series of 191 patients found infections in three-quarters of patients with both humoral and cellular arms impacted (58). Similarly to STAT3-HIES, data do not support a genotype-phenotype correlation and indeed, mutations affecting the same codon may cause both STAT3-GOF and STAT3-HIES diseases in different patients (58, 122).

Treatment is challenging, with incomplete response to individual agents and most patients require multiple (>5) lines of therapy to sustain a clinical response (58). The ability to target the JAK-STAT cascade using JAK inhibitors such as ruxolitinib provides an attractive treatment option in STAT3-GOF, with efficacious response although long-term data are lacking (58, 123, 124). Other targeted therapies include IL-6 blockade (tocilizumab) (123). The use of small molecules to inhibit STAT3 activity is the subject of several pre-clinical trials, focusing primarily on somatic STAT3-mutated malignancies (125). Whether these may translate to therapy for germline activating mutations remains to be seen. Data on outcomes of HSCT are limited. Initial reports were dismal, with death in 4/5 patients, though subsequent series have shown improved survival up to 62% in a summary of 23 transplanted patients (58, 126). HSCT in childhood may not normalize growth velocity. A more detailed exploration of indication, transplant strategy, and morbidity and mortality beyond individual case reports is yet to be published, particularly as HSCT appears to be reserved for treatment-resistant cases with significant pre-HSCT morbidity. The pleiotropic nature of STAT3 suggests that, as in STAT3-HIES, HSCT for STAT3-GOF may not reverse the signaling defect in extra-hematopoietic tissues.

### IL-2RA and IL-2RB deficiencies

Whilst deficiency of the γ-chain of the IL-2 receptor causes T-B+NK- SCID (127, 128), deficiency of the α or β chains, encoded by *IL2RA* and *IL2RB* respectively, cause immune dysregulation (129). The α chain (IL-2RA, CD25) exclusively binds IL-2, in contrast to the γ chain, which forms receptors that bind interleukins -4, -7, -9, -15, and -21. CD25 is expressed at high levels on Tregs, and binding of IL-2 upregulates FOXP3 expression and therefore enhances Treg activity in response to immune stimulus (130). Meanwhile, the β chain (IL-2RB, CD122) forms a receptor with the γ chain which binds IL-2 and IL-15 (131). IL2-RA and IL2-RB deficiencies are rare, with few published cases. Their shared phenotype includes autoimmunity, with erythroderma, enteropathy, and alopecia, similar to IPEX. However, in contrast, these patients also exhibit a significant susceptibility to herpesvirus infections such as CMV (12, 13, 57). Phosphorylation of STAT5 following IL-2 stimulation is reduced or absent, depending on whether the mutation is null or hypomorphic, in both syndromes (57). In IL-2RA deficiency, Treg numbers are low-normal, but the cells are non-functional; in IL-2RB deficiency, Tregs are low-absent (129). Autoreactive CD8+ cells may infiltrate organs causing autoimmunity in IL-2RA deficiency (12). Interestingly, some patients with hypomorphic IL-2RG mutations display a phenotype akin to IL-2RA and IL-2RB mutations, with immune dysregulation and lymphoproliferation (129).

HSCT has been reported in one patient with IL-2RA deficiency, who survived (132). In IL-2RB deficiency, two cases have been published and one additional, unpublished case was performed at our center, with survival and cure in two patients and death from CMV in the third (57). mTOR inhibition has been reported to improve autoimmune symptoms in one case (133). Pooling of data for these rare monogenic diseases is needed to better understand their manifestations and natural history.

### Primary and familial HLH syndromes

Hemophagocytic lymphohistiocytosis (HLH) describes a syndrome of immune hyperactivation caused by sustained activation of cytotoxic T-lymphocytes and resultant release of inflammatory cytokines, leading to persistent fever, hepatosplenomegaly, cytopenias, and coagulopathy (23). Whilst identification of hemophagocytes in bone marrow or cerebrospinal fluid specimens may aid diagnosis, absence does not exclude HLH (134). HLH may be classified by as primary (driven by monogenic IEI) or secondary (driven by environmental or acquired triggers, such as malignancy, infection, or inflammatory disease) (23). In the setting of rheumatological disease, typically systemic-onset juvenile idiopathic arthritis, HLH may be termed ‘macrophage activation syndrome.’ HLH is diagnosed using criteria defined by the Histocyte Society, including fever, splenomegaly, bicytopenia, hypertriglyceridemia and/or hypofibrinogenemia, hemophagocytes, low/absent NK-cell activity, hyperserotonemia and high soluble-IL-2-receptor levels; at least five criteria must be met in order to diagnose HLH (135). The treatment strategy set out by the HLH-2004 trial includes combination chemotherapy with etoposide, dexamethasone, ciclosporin A ± intrathecal methotrexate and corticosteroids (135, 136). In recent years, other therapeutic strategies have emerged, primarily as second-line or salvage therapy, including lymphodepletion with alemtuzumab (137, 138), blockade of inflammatory cytokines such as IL-1 (anakinra), IL-6 (tocilizumab), and IFN-γ (emapalumab) (43), and JAK-inhibition (ruxolitinib) (42, 139). Identification of whether HLH is primary or secondary is important, as patients with primary HLH should progress to allogeneic HSCT; this may rely on molecular or genetic studies as some infectious triggers, such as EBV, may cause either primary or secondary HLH.

Primary HLH may be subdivided into a collection of diseases impacting lymphocyte cytotoxicity, including the five ‘familial’ HLH (FHLH) syndromes where HLH is the primary disease manifestation, and those where impaired control of infection or dysregulated inflammasome activation leading to immune hyperactivation are seen in addition to other clinical manifestations. FHLH results from defective trafficking of vesicles containing cytotoxic molecules such as perforin and granzymes to a target cell, preventing its destruction and prolonging immune activation (140). Whilst the genetic aetiology of FHLH1 is unknown, the other syndromes relate to defects in vesicle content (FHLH2, *PRF1*), priming (FHLH3, *UNC132*), or docking and fusion to the target cell membrane (FHLH4, *STX11* and FHLH5, *STXBP2*) *(* 22, 140). The largest cohort published to date, from the HLH-2004 study, demonstrated 5-year overall survival of 71% in children with FHLH (141).

Aside from these familial syndromes, other IEI affecting lymphocyte granule-mediated cytotoxicity include pigmentary disorders such as Chediak-Higashi syndrome caused by mutations in *LYST*, Griscelli syndrome type 2, caused by *RAB27A* mutations, and less frequently in Hermansky-Pudlak syndrome type 2, caused by *AP3B1* mutations (142–144). Unfortunately, HSCT does not arrest the neurodevelopmental sequelae seen in Chediak-Higashi syndrome (145). Susceptibility to EBV-driven HLH may be seen in mutations of *SH2D1A*, causing X-linked lymphoproliferative syndrome (XLP) 1 and manifesting in hypogammaglobulinemia, and development of EBV-driven lymphoma, as well as other IEIs rendering patients unable to handle this virus effectively, such as deficiencies of CD27, CTPS1, ITK, and CD70 (146, 147). A multi-center study reporting the outcome of HSCT for XLP1 recommended that transplantation is undertaken in all patients who develop HLH, as the outcome without HSCT is poor (overall survival 18.8%) although HLH manifestations associated with higher mortality during HSCT (51). Autologous T-lymphocyte gene therapy is under investigation, with evidence of correction of the T-lymphocyte defect *in vitro (* 52). NK cell deficiency, such as that seen in *MCM4* defects, also predisposes to primary HLH due to inability to clear virus-infected cells (148, 149). Other genetic aetiologies of primary HLH include genes regulating inflammasome activity, such as *XIAP* and *NLRC4*. XIAP-deficient males may develop HLH following EBV infection in addition to splenomegaly and inflammatory bowel disease (150, 151). Whilst termed XLP2, unlike XLP1 there does not appear to be an increased risk of EBV-driven lymphoma, and HLH episodes may occur more frequently (152). The incidence of HLH in XIAP deficiency is approximately 60%, whilst half have splenomegaly and a quarter develop Crohn’s disease-like colitis. Allogeneic HSCT remains the only curative treatment for XIAP deficiency; whilst the odds of survival have improved drastically between patients transplanted before and after 2015 (41% versus 89%), outcomes are poorer following myeloablative conditioning and in patients with active HLH at HSCT. It is not fully understood whether patients with milder disease should be offered HSCT as the outcomes for patients managed conservatively vary (153). For XIAP patients with significant bowel inflammation, HSCT appears to resolve inflammatory bowel disease (154). Future targeted therapy to aid remission of autoinflammation in XIAP and NLCR4-related disease, which is driven by high IL-18 levels, may come in the form of tadekinig alfa, which mimics endogenous neutralizing IL-18 binding protein; whilst its use in XIAP deficiency has been reported in a single patient, further evidence on efficacy may come from a randomized trial (NCT03113760), which has completed recruitment and is ending soon (44, 155).

Whilst the genetic landscape of primary HLH has grown with increasing understanding of interactions leading to cell-mediated cytotoxicity, a high index of clinical suspicion is needed to identify possible HLH and institute appropriate initial management. Use of specialist investigations such as NK degranulation and perforin expression may shorten the time taken to differentiate primary from secondary HLH and allow earlier definitive therapy (156).

### PIRDs associated with VEOIBD

Children diagnosed with inflammatory bowel disease before the age of 6 years form a subset of patients that is enriched for underlying IEI (157). This group of patients may also be prone to failure of conventional immunomodulation, and for patients with underlying IEI the balance between immunosuppression to achieve symptom control versus infection risk may pose a challenge. Identified monogenic defects associated with VEOIBD include chronic granulomatous disease, IPEX syndrome, XIAP deficiency, Wiskott-Aldrich syndrome, and defects of IL-10 signaling. In 2009, mutations in *IL10RA* and *IL10RB* encoding the two subunits of the IL-10 receptor, were identified in four patients with VEOIBD; subsequent to this, loss-of-function mutations in IL-10 were also identified in association with intestinal inflammation (60, 158). This is consistent with previous findings showing IL-10 to be a key suppressor of T lymphocyte activation and effector function, particularly in the gastrointestinal tract (159). IL-10 defect-associated VEOIBD appears to be fully penetrant and presents with perianal disease alongside additional features such as folliculitis and arthritis. Whilst reported cases are few, allogeneic HSCT appears to be curative (60, 160, 161). Identification of a genetic mutation associated with VEOIBD is important, as not all monogenic causes relate to the hematopoietic system and thus allogeneic HSCT may not resolve VEOIBD caused by epithelial defects or tricho-hepatico-enteric syndromes (162, 163).

Loss of function mutations in *RIPK1*, encoding a serine/threonine-protein kinase involved in cell death regulation, also cause VEOIBD in tandem with combined immunodeficiency and arthritis, which may be rescued by allogeneic HSCT (164).

### Activated phosphoinositide-3 kinase δ syndrome

Activated phosphoinositide 3-kinase-δ syndrome (APDS) results from either gain-of-function mutations in *PIK3CD* encoding the catalytic subunit p110δ (APDS1), or by loss-of-function variants in *PIK3R1* encoding the regulatory subunits p85α, p55α, or p50α (APDS2) (165). APDS was first identified in 2013 in seven kindreds with recurrent respiratory infection progressing to bronchiectasis, and lymphopenia (166). The result of these dominant mutations is disruption of the tight balance in phosphoinositide 3-kinase (PI3K) activity, with hyperactivation of this pathway impacting T and B lymphocyte homeostasis (165). Patients with APDS manifest a heterogeneous immunophenotype, including reduced naïve and increased senescent CD8+ T-lymphocyte populations; reduced class-switched memory B-lymphocyte cells with increased transitional B-lymphocytes; and dysgammaglobulinemia, typically with elevated serum IgM with self-reactive antibody production but reduced IgA and IgG (167). Though not classified as a PIRD in the latest IUIS classification, this IEI shares many features with other PIRDs, as highlighted by the recent publication of an ESID registry study analyzing 170 patients (168). In addition to recurrent sinopulmonary infections, around 25% of patients have chronic EBV infection with high rates of lymphoproliferation (86%) and lymphoma (14%). AIC may occur, but less frequently than in CTLA-4 haploinsufficiency or STAT3-GOF, as with enteropathy and skin manifestations. Patients frequently develop bronchiectasis in childhood, and earlier onset of symptoms predicts worsening disease severity. Rare, but described manifestations include arthritis, pancreatic islet β-cell destruction causing diabetes, and neuroinflammation, which are all more common in other dominantly inherited PIRDs such as CTLA-4 haploinsufficiency. Phenotypically, APDS1 and APDS2 differ by the former being relatively enriched for AIC and skin disease, whilst the latter typically includes syndromic features such as short stature (167–169). Strategies to reduce infection frequency are antimicrobial prophylaxis and immunoglobulin replacement. Immune dysregulation may be treated with steroids, B-lymphocyte depleting agents, or mTOR inhibitors. mTOR inhibition is attractive given the high response rate of lymphoproliferation, enteropathy, and AIC, possibly explained by the hyperactivation of mTOR signaling downstream of Akt, which is enhanced by increased PI3K activity (168, 170). A retrospective study of 57 patients who underwent HSCT for APDS demonstrates the challenges in treating this disease with transplantation. Whilst 2-year overall survival probability was good (86%), there was a high rate of graft failure necessitating further cellular infusions, which rose from 10% at 1 year post-HSCT to 17% at 2 and 3 years after transplant (61). Strikingly, the incidence of graft failure was even higher in patients who received post-HSCT mTOR inhibitors, at 15-42% (1-3 years post-HSCT) compared to 9% in patients who did not receive mTOR inhibition, possibly due to giving remnant recipient lymphocytes a survival advantage over the reconstituting recipient cells (61). Targeted inhibition of PI3K with leniolisib is now possible, following first approval for treatment of APDS in adults and children aged 12 years and above in in the USA in 2023; the randomized controlled trial leading to its approval demonstrated high efficacy in reduction of lymphoproliferation and normalization of the APDS immunophenotype (49, 171). Further data, including exploration of leniolisib as a ‘bridge’ to HSCT, are warranted.

### PIRDs associated with autoinflammation

Systemic lupus erythematosus (SLE) has a strong heritable component indicated by the association of its phenotype with variants in multiple candidate genes in genome-wide association studies (172). In addition to population-level variation, several genes have been identified to correlate strongly with development of SLE in specific pedigrees despite low allele frequency in the general population, suggestive of a monogenic aetiology of SLE in this small subgroup. Consistent with the finding that DNA may act as an autoantigen in SLE, abnormal clearance of circulating DNA may cause tissue damage by provoking formation of autoantibodies. The primary enzyme mediating clearance of chromatin is DNase1. Low serum DNase1 activity is associated with development of autoimmune hepatitis, and higher SLE disease activity (173, 174). Monoallelic null mutations of *DNASE1* were found in two unrelated patients with SLE (175). Homozygous null mutations in *DNASE1L3*, preventing the expression of this protein which is closely related to DNase1, may also cause a fully-penetrant form of monogenic lupus (176).The role of deficiency of these enzymes in provoking autoimmunity suggests augmenting the activity of DNase may help neutralize errant chromatin and interfere with the pathogenesis of autoimmunity in murine models (177).

In 2016, heterozygous loss-of-function mutations in *TNFAIP3* leading to haploinsufficiency of its encoded protein, A20, were identified as a monogenic cause of Behçet disease (178). Haploinsufficiency of A20 (HA20) results in increased NFκβ signaling and subsequent upregulation of pro-inflammatory cytokines such as IL-1, IL-6, and TNFα; consequently, use of anakinra and anti-TNFα monoclonal antibodies such as infliximab are efficacious for controlling disease manifestations (178, 179). Symptoms of HA20 include Behçet disease-like features such as orogenital ulceration, arthritis, and uveitis. However, unlike polygenic Behçet disease, onset is typically in childhood and patients have recurrent febrile episodes (178, 179). HSCT has been reported in two patients with HA20, who both remitted completely (62).

IL-1 is a powerful inducer of fever and inflammation; upon binding of this cytokine to its receptor, transcription of pro-inflammatory cytokines is upregulated via MyD88, IRAK-4, and NFκβ (180). This process is regulated by IL-1RA, which competes with IL-1α and IL-1β for binding with the IL-1 receptor; recombinant IL-1RA (anakinra) is licensed for several autoimmune diseases including rheumatoid arthritis. Whilst impaired IL-1 results in immunodeficiency characterized by susceptibility to *Staphylococcus aureus*, *Streptococcus pneumoniae*, and *Pseudomonas aeruginosa* infections (181), deficiency of IL-1Ra leads to a syndrome of sterile multifocal osteomyelitis, periostitis, and pustulosis caused by unrestrained IL-1-mediated inflammation (DIRA) (47). These patients respond rapidly to replacement therapy with anakinra (47). Similarly, deficiency of IL-36RA, which shares 44% of its homology with IL-1RA, causes increased IL-1-related signaling and a clinical syndrome of infection-provoked generalized pustular psoriasis, fever and asthenia, which may progress to death from overwhelming infection (48, 182). This syndrome, labelled Deficiency of Interleukin-Thirty-six-receptor Antagonist, DITRA), does not appear to cause sterile osteoarticular inflammation seen in DIRA, but is responsive to anakinra (48).

## Future directions

The result of improved diagnostics and ability to differentiate patients with a PIRD phenotype into distinct genetic aetiologies is that understanding of how our immune system is regulated and how disruptions of this cause disease has grown, particularly over the past decade. Understanding these mechanisms has enabled development of targeted therapies that aim to ‘switch off’ a deleterious process whilst minimizing off-target effects. Many of these agents show significant promise in improving disease control and quality of life in patients with specific PIRDs, although long-term efficacy, and side effects are unknown. However, the inevitable conclusion of our ability to differentiate this patient group is that sample sizes are diluted, and our ability to fully understand the epidemiology, clinical and immunological phenotype, and treatment options for a given disease becomes more challenging and reliant on large, multicenter international efforts. Whilst time- and resource-consuming, these collaborations are necessary to further our understanding of these diseases, particularly for studies with a longitudinal component, such as assessing treatment efficacy or natural history. Setup and maintenance of prospective disease registries, including adaptations of existing projects such as addition of the IDDA2.1 score to the ESID registry, or formation of the German multi-organ AutoImmunity Network, www.g-a-i-n.de), will provide databases to be interrogated for future studies (27, 183).

Increasing attention has been placed on collection of quality-of-life data in patients with IEI, with recent publication of studies exploring the impact of chronic granulomatous disease, Wiskott-Aldrich syndrome, common variable immunodeficiency, and patients with SCID who have undergone HSCT (184–187). Whilst we intuitively understand that recurrent infection, hospitalization, and end-organ damage might negatively impact quality-of-life, it is important to explore associated factors, particularly when considering the impact of starting new treatments or offering HSCT. Few existing studies can relate to the diverse manifestations of PIRDs, and abstraction from studies that focus on a specific manifestation of immune dysregulation, such as colitis, do not account for the cumulative burden of having multiple organ system disease. Quality of life data pertaining to specific PIRDs are scant; one study of Finnish patients with APS1 identified poor general health and worsening fatigue and well-being with a longer interval from diagnosis to data collection (188). Use of quality-of-life instruments should form part of collaborative efforts to characterize clinical aspects of these diseases.

Intrinsic to discussions regarding HSCT in IEI is which patients should be offered HSCT, and at what stage of their disease course this treatment should be considered. For several IEI, the decision to offer HSCT early prior to accumulation of organ damage is justified by large multicenter studies, for example in SCID and chronic granulomatous disease (29, 189). However, for diseases with a variable natural history, or incomplete penetrance, the decision to offer HSCT exchanges a potential risk of subsequent poor health over a period of years-decades for a concentrated period of high risk around HSCT and during the initial period of immune reconstitution. Whilst the decision to undergo HSCT must come jointly from clinician and patients and their families, the potential risks and benefits of this treatment must be informed by good quality clinical data, requiring use of registries such as those coordinated by the European Blood and Marrow Transplant, European Society for Immunodeficiencies, and Stem Cell Transplantation for Immunodeficiencies in Europe groups.

## Conclusion

Conceptually, PIRDs provide a fascinating lens through which we can observe how alterations in immune regulatory genes lead to diverse clinical phenotypes. However, for patients, these diseases are far from fascinating. Their multi-organ manifestations may lead to delay in reaching a unifying diagnosis, and the chronicity of ill health, need for long-term treatment, and absence of natural history data for many, cast uncertainty on patient lives. Collective efforts to better understand these diseases and the therapies we may offer patients must continue.

## Author contributions

CT: Conceptualization, Resources, Writing – original draft, Writing – review & editing. MS: Conceptualization, Writing – review & editing. AG: Conceptualization, Writing – review & editing.
